# The relationship between epicardial adipose tissue and coronary artery stenosis by sex and menopausal status in patients with suspected angina

**DOI:** 10.1186/s13293-018-0212-8

**Published:** 2018-12-14

**Authors:** Mi-Na Kim, Seong-Mi Park, Dong-Hyuk Cho, Hack-Lyoung Kim, Mi-Seung Shin, Myung-A Kim, Kyung-Soon Hong, Wan-Joo Shim

**Affiliations:** 1 Division of Cardiology, Korea University Medical Center, Anam hospital, Seoul, South Korea; 2 Division of Cardiology, Seoul National University Boramae Medical center, Seoul, South Korea; 3 Division of Cardiology, Gil Medical Center, Gachon University College of Medicine, Incheon, South Korea; 4 Division of Cardiology, Hallym University College of Medicine, Chuncheon, South Korea; 5 Division of Cardiology, Department of Internal Medicine, Korea University Medical Center, Anam Hospital, Korea University College of Medicine, Inchonro 73, Seongbukgu, Seoul, 02841 South Korea

**Keywords:** Epicardial adipose tissue, Coronary artery stenosis, Women, Menopause

## Abstract

**Background:**

Evidence suggests that epicardial adipose tissue (EAT) is closely related to coronary artery stenosis (CAS). However, sexual dimorphism may be present in adipose tissue, and its influence on CAS between men and women is controversial. We assessed the relationship between EAT and CAS by sex and menopausal status in patients with suspected angina.

**Methods:**

Six hundred twenty-eight consecutive patients (men/women *n* = 257/371; mean age = 59.9 ± 10.2 years) who had chest pain for angina and underwent coronary angiography were included. CAS was defined as > 50% luminal narrowing of at least one epicardial coronary artery. EAT thickness was measured by transthoracic echocardiography.

**Results:**

Of the 628 patients, 52.1% (*n* = 134) of men and 35.3% (*n* = 131) of women had CAS. The mean EAT thickness was not different between men and women and was larger in patients with CAS (8.04 ± 2.39 vs 6.58 ± 1.88 mm, *P* < 0.001). EAT thickness was independently associated with CAS in both sexes (*P* < 0.001). The odds ratio (OR) of EAT for the presence of CAS was higher in men (OR = 1.43, 95% confidence interval [CI] 1.21–1.69) than in women (OR = 1.24, 95% CI 1.10–1.40). EAT thickness was larger in postmenopausal women than in premenopausal women (7.59 ± 2.25 vs 5.80 ± 1.57 mm, *P* < 0.001) and was independently related with CAS (OR = 1.24, 95% CI 1.09–1.41). This was not the case in premenopausal women.

**Conclusion:**

In patients with suspected angina, an increase in EAT thickness was independently related to the presence of CAS in both men and women, with it being stronger in men. According to menopausal status in women, EAT thickness is significantly associated with CAS only in postmenopausal women.

## Introduction

Growing evidence suggests that epicardial adipose tissue (EAT) is closely related to coronary artery stenosis (CAS). EAT is an emerging cardiometabolic risk factor due to the close proximity to the coronary artery and heart and its secretion of proatherogenic and proinflammatory adipokines [1]. EAT has a close relationship with metabolic syndromes, coronary atherosclerosis, and myocardial function [2–4].

Sexual dimorphism in adipose tissue deposits is well documented in humans [5]. Premenopausal women tend to accumulate subcutaneous adipose tissue, which is located primarily in the gluteal and femoral regions, whereas men accrue more visceral fat, which is closely related to increased metabolic and cardiovascular risk [5, 6]. After menopause, fat redistribution favoring visceral fat deposits occurs, and this shift leads to a parallel increase of cardiometabolic risk in women [7].

However, the sex difference of the association between EAT and CAS has not been thoroughly studied, and the results are still controversial. The menopausal transition in women is thought to be associated with changes in body fat deposition and composition rather than weight gain [8, 9]. The prevalence of EAT in postmenopausal women was greater than that of premenopausal women, independent of age, race, obesity, and other factors related to EAT [10, 11]. Therefore, the impact of EAT on cardiovascular structure and function in women could be different to menopausal status. The aim of this study was to evaluate the relationship between EAT and CAS by sex and menopausal status in patients with chest pain and are undergoing coronary angiography.

## Methods

### Study population

Six hundred twenty-eight consecutive patients (men/women = 257/371; mean age = 59.9 ± 10.2 years) were included for this study, who had visited the outpatient clinic for chest pain with suspected angina. This study was a part of the Korean Women’s Chest Pain Multicenter Registry data (KoROSE), which is a nation-wide multicenter registry, which is participating in 20 tertiary hospitals in Korea. Twenty- to 80-year-old patients presenting with chest pain, or other symptoms indicative of myocardial ischemia, were included if their coronary status had been evaluated [12]. Patients with previously confirmed coronary artery disease, significant valvular or structural heart disease, and significant medical diseases were excluded. This study protocol was reviewed and approved by the institutional review board in each center. The written informed consent for registration was obtained from each patient.

### Data collection

In each patient, laboratory tests, transthoracic echocardiography, and invasive coronary angiography (CAG) were performed within a 1-month interval. Hypertension, diabetes, and dyslipidemia were defined if the patient was previously diagnosed or had taken related medications. Furthermore, the patients were newly diagnosed with dyslipidemia if their lipid profiles met the criteria of dyslipidemia, according to the National Cholesterol Education Program Adult Treatment Panel III guideline [13]. Obesity was defined as a body mass index (BMI) ≥ 30. Menopausal status was assessed via a self-reported questionnaire. The invasive CAG was performed using a standard method in each patient. The severity of CAS was assessed by interventional cardiologists of each institute in a blind condition to the EAT data and concept of this study. Obstructive CAS was defined as the presence of > 50% luminal narrowing in at least one coronary artery.

### Measurement of EAT thickness

EAT thickness was measured with transthoracic echocardiography, using commercially available equipment (Vivid 7 and 9, General Electric Medical Health, Waukesha, WI; iE33, Philips Medical, Andover, MA). EAT thickness was measured as the maximal thickness of echo-free space from the right ventricular free wall to the visceral pericardium, in parasternal long-axis images at end-systole, using the aortic annulus as an anatomic landmark [14]. The intra-observer variability and inter-observer variability for EAT thickness measurement were excellent (interclass correlation coefficient [ICC] = 0.986, 95% confidence intervals [CI] 0.983–0.988, *P* < 0.001 and ICC = 0.898, 95% CI 0.788–0.951, *P* < 0.001, respectively).

### Statistical analysis

Quantitative data were presented as a mean ± standard deviation, and categorical data were expressed as numbers and percentages. To compare the two groups, a paired *t* test was used for continuous variables, and a chi-squared test was used for categorical variables. The analysis of covariance (ANCOVA) was used to compare mean EAT thicknesses, after adjusting for related factors of EAT that had been previously reported, including age, diabetes, hypertension, dyslipidemia, and BMI [15–17]. The estimated marginal mean of EAT thickness by ANCOVA was presented as a mean ± standard error, after adjusting for related factors of EAT. Univariate and multivariate binary logistic regression analyses were used to evaluate the relationship between coronary artery disease and the clinical risk factors and EAT. Also, to assess the sex difference’s effect of EAT on the presence of CAS, the interactive effect of sex × EAT thickness was examined using binary logistic regression. Age, BMI, and EAT thickness were used as continuous variables, and smoking, hypertension, diabetes, and dyslipidemia were used as categorical variables for regression analysis. The interactive plots were generated by ggplot2 R packages. The predictive value of EAT thickness for CAS was tested via receiver operating characteristic curve analysis. The cutoff point was selected using the value which had a maximum Youden’s index. Statistical significance was defined as a two-tailed *P* value of < 0.05 using 95% CIs. All analyses except the interactive plot were conducted using the SPSS for Windows version 20.0 (IBM, Armonk, NY, USA).

## Results

### Baseline characteristics

The baseline characteristics of patients are presented in Table 1. Men were younger than women. The prevalence of hypertension, diabetes, and dyslipidemia and a familial history of coronary artery disease were not significantly different between men and women. However, the prevalence of smoking was higher in men. In laboratory findings, the levels of low-density lipoprotein and triglycerides were higher in men, whereas the level of high-density lipoprotein was higher in women. The presence of CAS was more frequent in men than in women (*P* < 0.001).

**Table 1:** Baseline characteristics of the study population (*n* = 628)

	Men (*n* = 257)	Premenopausal women (*n* = 59)	Postmenopausal women (*n* = 312)	*P* value*
Age, years	57.6 ± 10.2^**§**^	47.3. ± 5.2	63.8 ± 8.5	< 0.001
BMI, kg/m^2^	24.8 ± 3.0	25.2 ± 3.6	25.2 ± 3.2	0.935
Waist, cm	85.9 ± 9.2^**§**^	72.9 ± 13.5	79.4 ± 9.0	< 0.001
Smoking, *n* (%)	88 (34.2%)^**§**^	4 (6.8%)	18 (5.8%)	0.765
Hypertension, *n* (%)	130 (50.6%)	13 (22.0%)	151 (48.4%)	< 0.001
Diabetes, *n* (%)	47 (18.3%)	4 (6.8%)	55 (17.6%)	0.034
Dyslipidemia, *n* (%)	57 (22.2%)	9 (15.3%)	73 (23.4%)	0.230
Obesity, *n* (%)	13 (5.1%)	6 (10.2%)	18 (5.8%)	0.224
Family Hx of CAD, *n* (%)	47 (18.2%)	6 (10.2%)	61 (20.7%)	0.096
Coronary angiographic finding				
CAS, *n* (%)	134 (52.1%)^**§**^	10 (16.9%)	121 (38.8%)	0.001
Medication				
ACEi or ARB, *n* (%)	73 (28.5%)	8 (13.3%)	84 (26.9%)	0.033
CCB, *n* (%)	79 (30.9%)	14 (23.3%)	93 (29.8%)	0.353
Beta-blocker, *n* (%)	36 (14.1%)	3 (5.0%)	46 (14.7%)	0.039
Statins, *n* (%)	38 (14.8%)	2 (3.3%)	38 (12.2%)	0.041
Anti-platelet agent, *n* (%)	68 (26.6%)^**§**^	18 (30.0%)	109 (34.9%)	0.552
Laboratory finding				
Glucose, mg/dL	114.0 ± 36.2	127.1 ± 130.4	107.4 ± 23.9	0.347
Total cholesterol, mg/dL	179.8 ± 42.2	190.5 ± 40.1	185.8 ± 44.7	0.501
HDL, mg/dL	46.2 ± 30.2^**§**^	50.8 ± 10.8	52.3 ± 14.9	0.428
LDL, mg/dL	122.9 ± 34.0^**§**^	110.6 ± 33.5	112.0 ± 34.1	0.580
Triglyceride, mg/dL	169.0 ± 108.6^**§**^	126.8 ± 71.2	137.6 ± 125.8	0.440
hs-CRP, mg/dL	2.20 ± 4.20	1.16 ± 1.32	2.59 ± 7.30	0.040

*BMI* body mass index, *Hx of CAD* history of coronary artery disease, *CAS* coronary artery stenosis, *ACEi* angiotensin-converting enzyme inhibitor, *ARB* angiotensin receptor blocker, *CCB* calcium channel blocker, *WBC* white blood cell, *LDL* low-density lipoprotein, *HDL* high-density lipoprotein, *hs-CRP* high sensitive C-reactive protein

*Comparison between pre- and postmenopausal women

^**§**^*P* value < 0.05 comparison between men and women

In women, 312 patients (84.1%) were postmenopausal. Among them, only 7 patients (2% of postmenopausal women) took hormonal replacement therapy. Although BMI was not significantly different between pre- and postmenopausal women, waist circumference was larger in postmenopausal women. The prevalence of hypertension and diabetes was higher in postmenopausal women. In laboratory findings, lipid profiles were not different between pre- and postmenopausal women, but high sensitivity C-reactive protein (hs-CRP) levels were higher in postmenopausal women than in premenopausal women (*P* = 0.04). The presence of CAS was more prevalent in postmenopausal women than in premenopausal women (*P* = 0.001).

### Comparison of the relationship between EAT thickness and CAS by the sex

In a total of 628 patients, the mean EAT thickness was 7.21 ± 2.23 mm. EAT thickness weakly, but positively, correlated with age (*r* = 0.28, *P* < 0.001). EAT thickness was larger in patients with hypertension (7.66 ± 2.34 vs 6.80 ± 2.05 mm, *P* < 0.001) and in patients with diabetes (7.81 ± 2.38 vs 7.08 ± 2.18 mm, *P* = 0.002) but not in patients with dyslipidemia (*P* = 0.169). BMI had a weak relationship with EAT thickness (*r* = 0.12, *P* = 0.003).

**Fig. 1:**
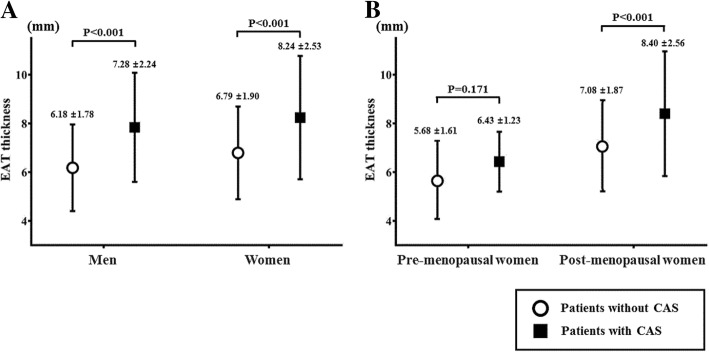
The difference in EAT thickness by the presence of CAS. **a** The difference of EAT thickness between patients with and without CAS in both men and women. **b** The difference of EAT thickness between patients with and without CAS in pre- and postmenopausal women. CAS, coronary artery stenosis

EAT thickness was larger in patients with CAS than in patients without CAS (8.04 ± 2.39 mm vs 6.58 ± 1.88 mm, *P* < 0.001). An analysis according to dividing sex, EAT thickness was not different between men and women (7.05 ± 2.20 vs 7.31 ± 2.25 mm, *P* = 0.155) and was also larger in patients with CAS in both men and women (Fig. 1a). Even after adjusting for age, diabetes, hypertension, and BMI, EAT thickness was larger in patients with CAS than in patients without CAS in all patients and in each sex (Table 2).

**Table 2:** The comparison of EAT thickness (mm) between patients with and without CAS

	Before adjustment			After adjustment		
	Patients without CAS	Patients with CAS	*P* value	Patients without CAS	Patients with CAS	*P* value*
Total population	6.59 ± 1.88	8.04 ± 2.39	< 0.001	6.70 ± 0.12	7.76 ± 0.14	< 0.001
Men	6.18 ± 1.78	7.84 ± 2.24	< 0.001	6.07 ± 0.22	7.52 ± 0.20	< 0.001
Women	6.79 ± 1.90	8.24 ± 2.53	< 0.001	6.98 ± 0.14	7.90 ± 0.19	< 0.001
Premenopause	5.68 ± 1.61	6.43 ± 1.23	0.171	5.70 ± 0.21	6.31 ± 0.47	0.244
Postmenopause	7.08 ± 1.87	8.40 ± 2.56	< 0.001	7.21 ± 0.16	8.19 ± 0.20	< 0.001

The values were presented as mean ± standard deviation in the comparison between patients with and without CAS before adjustment and estimated marginal mean ± standard error presented for the values in the comparison between patients with and without CAS after adjustment

*EAT* epicardial adipose tissue, *CAS* coronary artery stenosis, *SE* standard error

*P value after adjustment factors that are related with EAT thickness, like age, diabetes, hypertension, and body mass index

**Fig. 2:**
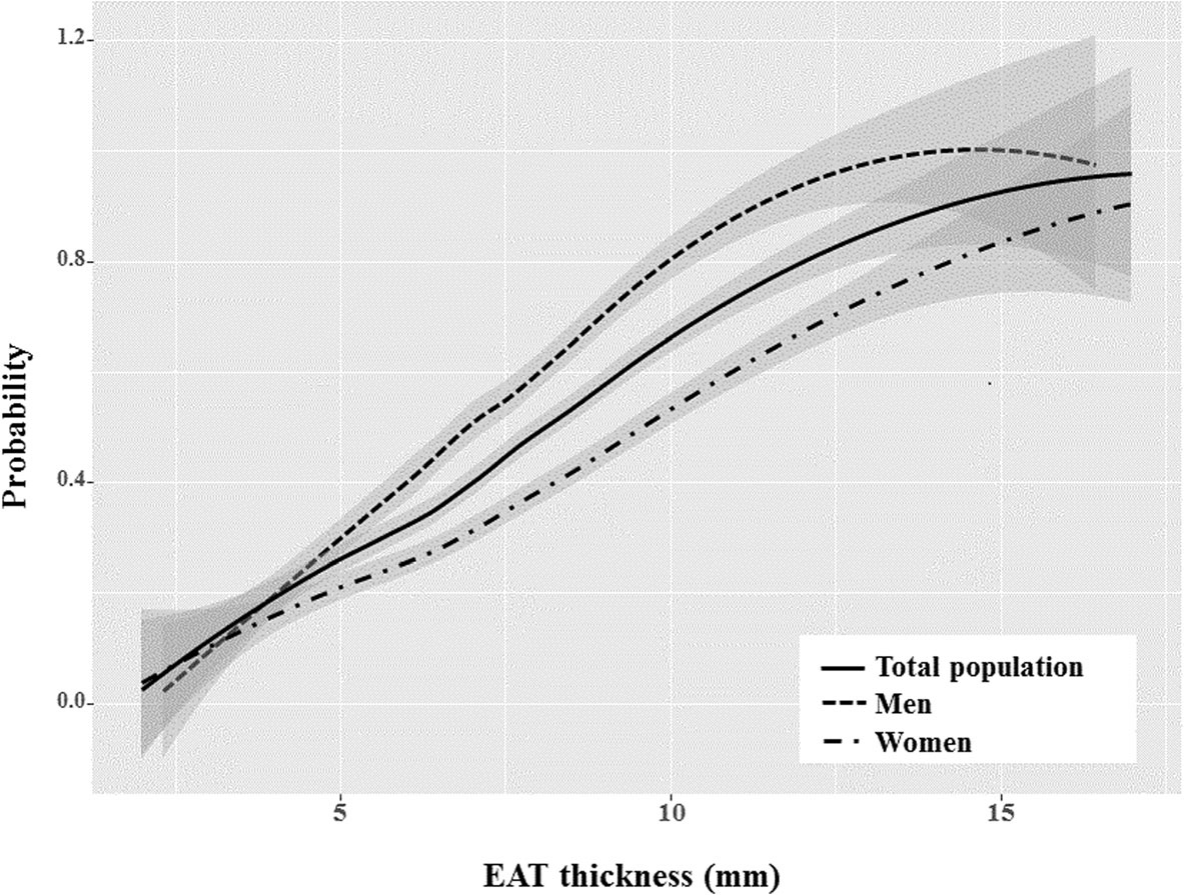
The interactive plot for the sex difference of the relationship between EAT and CAS

Age, diabetes, and EAT thickness were associated with CAS by univariate analysis in both sexes. Dyslipidemia was associated with CAS only in men, whereas hypertension was associated only in women by univariate analysis. Using multivariate analysis, EAT thickness was independently associated with CAS in both sexes (Table 3). However, the impact of EAT on the presence of CAS in men was greater than that in women by interaction analysis (Fig. 2).

**Table 3:** The relationship of CAS to CV risk factors and EAT thickness

	Men (*n* = 257)						Women (*n* = 371)					
	Univariate			Multivariate			Univariate			Multivariate		
	OR	95% CI	*P* value	OR	95% CI	*P* value	OR	95% CI	*P* value	OR	95% CI	*P* value
Age	1.05	1.02–1.08	0.001	1.04	1.01–1.08	0.020	1.07	1.04–1.10	< 0.001	1.05	1.02–1.08	0.001
BMI	1.09	1.00–1.01	0.060	1.07	0.96–1.21	0.227	0.99	0.92–1.05	0.669	0.95	0.87–1.02	0.161
Smoking	1.40	0.84–2.35	0.199	1.13	0.57–2.21	0.733	1.31	0.54–3.15	0.547	1.14	0.53–3.90	0.481
Hypertension	1.57	0.96–2.57	0.072	1.08	0.55–2.09	0.829	2.64	1.70–4.08	< 0.001	1.04	0.59–1.82	0.895
Diabetes	2.26	1.16–4.42	0.017	2.29	0.96–5.46	0.061	5.11	2.81–9.30	< 0.001	3.70	1.94–7.10	< 0.001
Dyslipidemia	2.03	1.11–3.70	0.022	2.63	1.10–6.25	0.028	1.23	0.74–2.04	0.331	1.02	0.58–1.79	0.948
EAT thickness	1.52	1.31–1.75	< 0.001	1.43	1.21–1.69	< 0.001	1.36	1.22–1.52	< 0.001	1.24	1.10–1.40	< 0.001

*CAS* coronary artery stenosis, *CV* cardiovascular, *OR* odd ratio, *CI* confidence interval, *BMI* body mass index, *EAT* epicardial adipose tissue

### Difference in the relationship between EAT thickness and CAS by menopausal status

The EAT thickness of postmenopausal women was larger than that of premenopausal women (7.59 ± 2.25 vs 5.80 ± 1.57 mm, *P* < 0.001). This trend remained after adjusting for age, diabetes, hypertension, and BMI (7.43 ± 0.12 mm, 95% CI 7.19–7.67 vs 6.68 ± 0.33 mm, 95% CI 6.04–7.32, *P* = 0.041). In postmenopausal women, EAT thickness, age, hypertension, and diabetes were related to CAS by univariate analysis (Table 4). EAT thickness was larger in postmenopausal women with CAS than those without CAS (Fig. 1b), and this trend persisted after adjusting for age, diabetes, hypertension, and BMI (Table 2). The multivariate analysis also showed that EAT thickness was independently associated with CAS in postmenopausal women (OR 1.244, 95% CI 1.09–1.41, *P* = 0.001).

**Table 4:** The relationship of CAS and CV risk factors and EAT in pre- and postmenopausal women

	Premenopausal women (*n* = 59)						Postmenopausal women (*n* = 312)					
	Univariate			Multivariate			Univariate			Multivariate		
	OR	95% CI	*P* value	OR	95% CI	*P* value	OR	95% CI	*P* value	OR	95% CI	*P* value
Age	1.09	0.94–1.27	0.401	1.13	0.94–1.35	0.250	1.07	1.04–1.10	< 0.001	1.05	1.02–1.09	0.004
BMI	1.03	0.85–1.25	0.720	1.00	0.80–1.24	0.978	0.98	0.91–1.05	0.551	0.93	0.85–1.01	0.095
Smoking	5.88	0.72–47.9	0.094	29.9	1.15–78.3	0.083	1.02	0.39–2.72	0.943	1.06	0.35–3.23	0.926
Hypertension	1.67	0.37–7.65	0.587	1.59	0.26–9.69	0.698	2.47	1.55–3.94	< 0.001	1.73	1.02–2.96	0.038
Diabetes	1.70	0.16–18.3	0.647	1.69	0.93–21.6	0.803	5.17	2.75–9.85	< 0.001	4.38	2.20–8.74	< 0.001
Dyslipidemia	1.76	0.19–15.9	0.542	7.17	0.30–26.3	0.251	1.22	0.72–2.08	0.411	1.21	0.47–2.19	0.528
EAT thickness	1.40	0.86–2.28	0.177	1.58	0.86–2.90	0.140	1.32	1.18–1.48	< 0.001	1.24	1.09–1.41	0.001

*CAS* coronary artery stenosis, *CV* cardiovascular, *OR* odd ratio, *CI* confidence interval, *BMI* body mass index, *EAT* epicardial adipose tissue

However, in premenopausal women, there was no difference in EAT thickness between patients with and without CAS (Table 2), and EAT thickness was not related to CAS. Only a history of smoking was associated with CAS in premenopausal women (Table 4). In comparison to age-matched men (mean age = 47.5 ± 5.9 years old), EAT thickness was larger in men than in premenopausal women (6.73 ± 2.11 mm vs 5.80 ± 1.57 mm, *P* = 0.008). In these men, EAT thickness was larger in patients with CAS than patients without CAS.

## Discussion

The main findings of this study were as follows: (1) EAT thickness was similar between men and women and was closely related with the presence of CAS in both sexes, but a stronger relationship was shown in men. (2) When analyzed by menopausal status in women, EAT thickness was one of the independent parameters relating to the presence of CAS in postmenopausal women but not in premenopausal women.

Based on previous studies, it was generally accepted that EAT has a direct role in the development and progression of coronary atherosclerosis [1, 3]. We also demonstrated that EAT thickness has a significant association with CAS, as well as severity [16]. An increased amount of EAT would influence the structure and function of the coronary artery by altering the balance of secreting cytokines towards pro-atherogenic and pro-inflammatory conditions via the paracrine and vasocrine routes [18, 19]. In previous studies, a significant relationship between EAT amount and coronary artery calcification was shown in men but not in women [20–22]. By contrast, this sex difference in relation to EAT with coronary artery calcification was not demonstrated in large cohort studies [23, 24]. Dagvasumberel et al. also reported that an increased EAT volume was associated with CAS (luminal narrowing over 50%) in men only [25]. However, in this study, EAT thickness was independently associated with CAS in women, as well as in men. According to menopausal status, EAT was associated with CAS only in postmenopausal women. Therefore, an independent relationship of EAT with CAS in all women may be reflected by the result in postmenopausal women, as the majority of women were of postmenopausal status (84%).

The reasons for the difference are unclear, but a possible cause is the difference of sexual dimorphism in EAT during aging. Experimental data revealed that EAT function decreased with aging in female rats but was relatively unchanged with age in male rats [26, 27]. These differences might help us to understand the sex differences of the relationship of EAT and CAS and change of these associations after menopause in women. Another possible cause is sex hormonal status. Estrogen affects body fat composition and energy expenditure. Through menopausal transition, women undergo an increase in visceral adipose tissue and a decrease in energy expenditure [28, 29]. Both subcutaneous adipose tissue and visceral adipose tissue express estrogen receptor (ER)α and ERβ. ERα plays an important role in the activity of adipocytes and sexual dimorphism of fat distribution. However, the effects of estradiol (E2) on lipolysis and lipid accumulation are different in specific regions of the body [29]. E2 inhibits lipolysis in subcutaneous adipocytes by increasing the expression of α2A-adrenergic receptors via ERα [30, 31], but this effect was much smaller in visceral adipocytes [32]. Therefore, lower levels of estrogen might lead to increased amounts of visceral adipose tissue, including EAT, in men and postmenopausal women. In addition, EAT is regarded as white adipose tissue, but recent studies reported that EAT has characteristics of brown or beige adipose tissue. Sacks et al. demonstrated that EAT has high expression of mitochondrial uncoupling protein-1 (UCP-1), which is the main marker of brown adipose tissue [33]. Estrogen could increase the gene expression of UCP-1 [34] and influence mitochondriogenesis via the mitochondrial ERα of brown adipose tissue [35]. Therefore, the feature of EAT as brown adipose tissue, which was associated with a more favorable effect on cardiovascular disease [36, 37], might be relatively small in men and diminish in postmenopausal women. Finally, as estrogen is associated with decreased expression of pro-inflammatory adipokines, such as tumor necrosis factor-alpha and interleukins [29], postmenopausal women tend to change to inflammatory conditions. Therefore, it is inferred that the decrement of estrogen might be one of the possible cause of increasing EAT thickness, which is related to CAS in postmenopausal women.

In this study, EAT thickness was more relevant to CAS in men than in women even with a similar EAT thickness between men and women. The presence of CAS was also lower in women than in men, which was consistent with other previous studies [16, 38]. It is known that ischemia but no obstructive coronary artery disease (INOCA) is more prevalent in women, and they often have coronary microvascular dysfunction [39, 40]. Therefore, a considerable number of women with INOCA should be classified as no CAS, and the power of EAT thickness might be attenuated.

The present study has several limitations. As this study was cross-sectional, it was impossible to demonstrate a cause-effect relationship between EAT and CAS. We did not measure serum markers such as adipokines and inflammation markers, except hs-CRP. In this study, hs-CRP was not statistically different between men and women, but postmenopausal women had higher hs-CRP than premenopausal women. Another limitation was that menopausal status had been assessed by self-questionnaire. The measurement of E2 levels had not been performed, and the direct relation between estrogen and EAT had not been conducted. Finally, EAT amount was assessed by the thickness on echocardiography. Even though EAT thickness measured by echocardiography correlated with EAT volume by computed tomography [41], it might be less accurate. However, echocardiography has the virtue of lower cost and no radiation exposure compared to the other measurement techniques, and it could also be easily performed at bedside.

## Conclusion

An increase in EAT thickness was independently related to the presence of CAS in both men and women, with it being stronger in men. According to menopausal status in women, EAT thickness is significantly associated with CAS only in postmenopausal women. From these results, we could show the different relationships of EAT to CAS between men and women, and the assessment of EAT thickness would be helpful to estimate the risk of atherosclerotic coronary artery disease in patients with suspected angina.
